# PET imaging of [52 Mn]Mn-DOTATATE and [52 Mn]Mn-DOTA-JR11

**DOI:** 10.21203/rs.3.rs-4684098/v1

**Published:** 2024-08-09

**Authors:** James M. Omweri, Hailey A. Houson, Shannon E. Lynch, Volkan Tekin, Anna G. Sorace, Suzanne E. Lapi

**Affiliations:** University of Alabama at Birmingham; University of Alabama at Birmingham; University of Alabama at Birmingham; University of Alabama at Birmingham; University of Alabama at Birmingham; University of Alabama at Birmingham

**Keywords:** agonists/antagonists, peptides, AR42J, manganese-52, SSTR2

## Abstract

Manganese-52 is gaining interest as an isotope for PET imaging due to its desirable decay and chemical properties for radiopharmaceutical development. Somatostatin receptor 2 (SSTR2) is significantly overexpressed by neuroendocrine tumors (NETs) and is an important target for nuclear imaging and therapy. As an agonist, [^68^Ga]Ga-DOTATATE has demonstrated significant internalization upon interaction with receptor ligands, whereas [^68^Ga]Ga-DOTA-JR11(as an antagonist) exhibits limited internalization but better pharmacokinetics and increased tumor uptake. The goal of this study was to label both DOTATATE and DOTA-JR11 peptides with ^52^Mn in high radiochemical yields (RCY) and sufficient specific activity. A comparison of these two compounds was performed in *in vitro* and *in vivo* studies in animals with somatostatin receptor-positive xenografts to characterize differences in cell, tumor, and tissue uptake.

Radiolabeling of DOTATATE and DOTA-JR11 was carried out by combining varying concentrations of the peptides with [^52^Mn]MnCl_2_. *In vitro* stability of the radiotracers was determined in mouse serum. *In vitro* cell uptake and internalization assays were performed in SSTR2 + AR42J cells and negative controls. *In vivo* biodistribution and longitudinal PET imaging was evaluated in mice bearing AR42J tumors.

Both [^52^Mn]Mn-DOTATATE and [^52^Mn]Mn-DOTA-JR11showed affinity for SSTR2 in AR42J cells. However, the uptake of [^52^Mn]Mn-DOTATATE was higher (11.95 ± 0.71%/ mg) compared to [^52^Mn]Mn-DOTA-JR11 (7.31 ± 0.38%/ mg) after 2 h incubation. After 4 h incubation, 53.13 ± 1.83% of the total activity of [^52^Mn]Mn-DOTATATE was internalized, whereas only 20.85 ± 0.59% of the total activity of [^52^Mn]Mn-DOTA-JR11 was internalized. The PET images revealed similar biodistribution results, with [^52^Mn]Mn-DOTATATE showing a significant tumor uptake of 11.16 ± 2.97% ID/g, while [^52^Mn]Mn-DOTA-JR11 exhibited a lower tumor uptake of 2.11 ± 0.30% ID/g 4 h post-injection.

The synthesis of both radiotracers was accomplished with high RCY and purity. The cell uptake and internalization of [^52^Mn]Mn-DOTATATE showed higher levels compared to [^52^Mn]Mn-DOTA-JR11. PET images of the radiotracers in AR42J tumor bearing mice demonstrated similar biodistribution in all organs except the tumor, with [^52^Mn]Mn-DOTATATE showing higher tumor uptake compared to [^52^Mn]Mn-DOTA-JR11. The variations in properties of these tracers could be used to guide further imaging and treatment studies.

## 1.0 Introduction

Radiolabeled peptides with positron-emitting radiometals including ^68^Ga, ^86^Y, and ^64^Cu have been used in targeted Positron Emission Tomography (PET) imaging of SSTR2, a receptor highly expressed in neuroendocrine tumors (NETs) [1–4]. Since the Food and Drug Administration (FDA), approved [^68^Ga]Ga-DOTATATE in 2016, followed by [^64^Cu]Cu-DOTATATE in 2020, SSTR2 PET/CT imaging has become standard of care for detecting, staging, and restaging NETs [1, 5, 6]. Furthermore, SSTR2 imaging offers valuable insights into identifying patients who could potentially benefit from peptide receptor radionuclide therapy (PRRT) with SSTR2-targeted radiopharmaceuticals including [^177^Lu]Lu-DOTATATE. DOTATATE, DOTATOC, and DOTANOC agents are all SSTR agonists where the mode of action involves internalization into tumor cells following the interaction between the ligand and the receptor [6, 7]. Conversely, SSTR antagonists do not undergo internalization into tumor cells upon receptor interaction, hence they were not initially seen as suitable targeting agents for the development of PET radiopharmaceuticals [4, 5, 7–10]. However, preclinical studies conducted by Ginji et al. among others, revealed that the radiolabeled SSTR antagonists can exhibit superior tumor-targeting capabilities compared to the agonists which is attributed to the ability of antagonists to identify and attach to a greater number of binding sites on the receptors [11]. Similar findings have been reported by various research groups that have investigated the impact of chelator, radiometal, and tumor type on tumor uptake [8, 10, 12].

Manganese-52, ^52^Mn (t_1/2_ = 5.6 d, β^+^ = 29.6%, E_βave_ = 242 keV) possesses favorable decay and chemical characteristics for PET imaging, especially in the development of long-lived PET radiopharmaceuticals. The 5.6-day half-life allows for enough time for the separation of the target, the process of radiochemistry, distribution to other sites, and PET imaging for several days following injection. The decay process includes a positron decay branching ratio of 29.6% and emits positrons with an average energy of 242 keV, which is comparable to that of ^18^F (250 keV) [13–17]. This results in high-resolution PET images.

Mn^2^+ is a hard Lewis acid and exhibits a preference for coordination with hexadentate or heptadentate ligands containing N and O as donor atoms [13]. The chelator DOTA has been successfully radiolabeled with ^52^Mn and has demonstrated stability both *in vitro* and *in vivo* [14]. In this current study, we aimed to assess and compare the targeting ability of [^52^Mn]Mn-DOTA-TATE (agonist) and [^52^Mn]Mn-DOTA-JR11(antagonist) in both *in vitro* and *in vivo* using PET imaging.

## 2.0 Materials and methods

Natural chromium powder (5 N purity) was purchased from ESPI metals (Ashland, OR). 1 mL SPE tubes with frits were sourced from Millipore Sigma (Burlington, MA). AG1-X8 analytical grade 200–400 mesh chloride form resin was obtained from Bio-Rad (Hercules, CA). DOTATATE was obtained from Macrocyclics (Plano, TX) while DOTA-JR11 was obtained from CPC scientific (San Jose, CA). Mouse serum was sourced from EMD Millipore Corporation (Temecula, CA). Silica-embedded iTLC-SG paper was obtained from Sorbtech Technologies (Norcross, GA). A549, MCF7, and AR42J cells were all purchased from the American Type Culture Collection (Manassas, VA). All other chemicals and solvents were purchased from Fisher Scientific (Hampton, NH) unless otherwise specified.

### 2.1 Production and purification of ^52^Mn

^52^Mn was produced by irradiation of natural chromium targets on a TR24 cyclotron (Advanced Cyclotron Systems Inc.) through the ^nat^Cr(p,n)^52^Mn nuclear reaction, as described in previous studies [17, 18]. For purification, AGI-X8 resin packed in 1 mL solid-phase extraction (SPE) tubes was used, and the elution of [^52^Mn]MnCl_2_ was carried out in 6 M HCl as previously reported by Pyles et al. [17].

### 2.2 Radiolabeling of DOTATATE and DOTA-JR11 with ^52^Mn

DOTATATE and DOTA-JR11 were dissolved in water to make stock solutions at a concentration of 1 mg/mL. 3.7 MBq (100 μCi) of ammonium acetate buffered [^52^Mn]MnCl_2_ (pH 4.5) was added to a 1.5 mL microcentrifuge tube containing 100 μL of 0.25 M ammonium acetate, pH 4.5, and varying concentrations of the ligands (0.1–1.0 nM). The reaction mixtures were incubated at 90°C while shaking for 30 min on a thermomixer (800 RPM). The radiochemical yields (RCY) of the radioligands were assessed using radio-TLC (instant thin layer chromatography) by using silica-embedded iTLC-SG paper. The iTLC paper was developed in 0.1 M EDTA, pH 5 as the mobile phase and scanned using an AR-2000 Imaging Scanner (Eckert and Ziegler, MA, USA). Additionally, radio-HPLC (high-performance liquid chromatography) using a HyPURITY^™^ C18 HPLC Column,150 × 4 mm, 5 μm particle size from Thermo Scientific (Waltham, MA, USA) was used to characterize the two compounds with a gradient elution. HPLC was performed on an Agilent 1260 system (Santa Clara, CA, USA) with an in-line Flow-RAM NaI detector (LabLogic, Brandon, FL, USA) with eluents, A = 0.1% trifluoroacetic acid in water and B = 0.1% trifluoroacetic acid in acetonitrile. The gradient elution was as follows: 0–2 min 95% A, 2–22 min 95% A → 5% A, 22–24 min 5% A, 24–26 min 5% A → 95% A, 26–30 min 95% A; flow rate: 1 mL/min.

### 2.3 Stability of the radioligands in mouse serum

For serum stability evaluation, 50 μL each of [^52^Mn]Mn-DOTATATE and [^52^Mn]Mn-DOTA-JR11 were separately mixed with 500 μL of mouse serum in triplicate and incubated at 37°C. At the specified time points, 40 μL of serum/radioligand mixture was collected and combined with an equal amount of methanol to separate serum proteins, as described in a previously published protocol on metabolite extraction and protein removal [19]. The supernatant was analyzed using both radio TLC and radio HPLC, as described above to determine the percentage intact complex.

### 2.4 Cell studies

#### 2.4.1 Cell culture

A549 (human lung cancer) and MCF-7 (human breast cancer) cell lines were grown in Gibbco’s high glucose Dulbecco’ Modified Eagle’s Medium (DMEM) that was supplemented with 10% fetal bovine serum (FBS) and 80 μM gentamicin. AR42J (rat pancreatic carcinoma) cell line was cultured in Roswell Park Memorial Institute (RPMI) 1640 media containing 20% FBS and 1 μg/mL gentamicin. The cells were maintained and grown in humidified incubators at 37°C with 5% CO_2_ atmosphere.

#### 2.4.2 Flow cytometry

Flow cytometry experiments were conducted to determine levels of SSTR2 expression. For fluorescence-associated cell sorting (FACS), MCF-7 and A549 cells were isolated from culture using 0.25% Trypsin and cell viability was assessed via Trypan blue exclusion method. Samples (n = 3 per cell line) were resuspended at 1×10^6^ cells per mL of 1X FACS buffer (1% bovine serum albumin in phosphate-buffered saline), and subsequently incubated with fluorophore-conjugated antibodies for 30 min in the dark at 4 °C. Antibodies utilized were 1 μg SSTR2 (Novus, #MAB4224) conjugated with Cy5.5 (LumiProbe, 7321-10rxn), and Near-IR Dead Cell Stain Kit (ThermoFisher, L34975). Data was acquired on an Attune NxT flow cytometer obtained from ThermoFisher Scientific (Waltham, MA, USA) and subsequent analysis was performed in FlowJo 10.6.2 software. Analysis of relative SSTR2 expression was determined using mean fluorescent intensity (MFI) values, frequency, and count of SSTR2 + populations.

#### 2.4.3 Cell uptake

1×10^5^ AR42J, A549, and MCF-7 cells were seeded onto 24-well plates (n = 6) and incubated at 37°C 48 h before study. The incubating cell media was removed followed by addition of 1 mL of fresh media containing 0.5 nM of either [^52^Mn]Mn-DOTATATE or [^52^Mn]Mn-DOTA-JR11 with a specific activity of ~ 0.68 ± 0.05 MBq/nmol (18.31 ± 1.05 μCi/nmol) into each well. The cells were incubated at 37°C under 5% CO_2_ for 2 h. Following incubation, the media was removed, and the cells were washed 3 times with ice-cold PBS. 500 μL of 0.2 M NaOH was added to detach the cells before collection into microcentrifugetubes followed by a 500 μL wash of PBS. Associated radioactivity was counted on a HIDEX (Turku, Finland) AGM gamma counter.

To normalize the counts to the total protein amount, a BCA protein assay (ThermoFisher Scientific) was performed, and the final data reported in % cell uptake/mg of protein.

#### 2.4.4 Cell internalization

Following a published procedure with slight modifications [20], 5× 10^5^ AR42J and MCF7 cells were seeded to 12-well plates and incubated overnight at 37°C under 5% CO_2_. After removing the culture media, 1 μL of fresh media containing 0.5 nM of either [^52^Mn]Mn-DOTATATE or [^52^Mn]Mn-DOTA-JR11 with specific activity of 0.37 MBq/μg was added to each well and the plates were incubated at 37°C under 5% CO_2_ for various time points (30 min, 1, 2, 4, and 24 h). At each time point, the media was removed, and cells were washed 3 times with ice-cold PBS.

To determine the surface bound fraction, 0.5 mL of 0.1 M citric acid (pH 5) was added to each well and incubated for 5 min. The fraction was collected into a microcentrifuge tube followed by addition of 0.5 mL gentle wash of PBS. To determine the internalized activity, 0.5 mL of 0.2 M NaOH was added to each well and incubated at 37°C for 5 min. This fraction was collected in a separate tube followed by addition of 0.5 mL of PBS wash. The final results were expressed as a percentage of the total activity that was present in each of the two fractions.

### 2.5 Animal studies

All animal studies were conducted using a protocol approved by the Institutional Animal Care and Use Committee (IACUC) at the University of Alabama at Birmingham and were compliant with national animal welfare policies and guidelines.

Female athymic nude mice purchased form Charles River lab (Charles River, Wilmington, MA) were left for one week to acclimate before initiating experiments.

#### 2.5.1 Tumor implantation

Subcutaneous tumor xenograft models for AR42J were established by implanting 1×10^6^ cells in complete cell media subcutaneously into the right shoulder 3–4 weeks (tumor average diameter of between 5–7 mm) before study.

#### 2.5.2 PET/CT imaging and biodistribution

Approximately 2.22 ± 0.20 MBq (60.0 ± 5.4 μCi) of [^52^Mn]Mn-DOTATATE or [^52^Mn]Mn-DOTA-JR11 (6 μg) in 100 μL injection doses in saline were prepared. Mice (n = 4 per group) were anesthetized with 2.5% isoflurane in oxygen and were injected via the retroorbital sinus. After injection, at 4, 24, and 48 h, mice were imaged on a Sofie GNEXT PET/CT small animal scanner (Sofie Biosciences, Dulles, VA, USA). At each time point, 30 min of PET data were acquired followed by a 3-min CT at 80 kVp for anatomical reference. At each time point post imaging, mice were euthanized, and organs were collected, weighed, and counted for associated radioactivity on gamma counter. Radioactivity uptake was calculated as the percent injected dose per gram of tissue (% ID/g).

For data processing, PET images were reconstructed *via* 3D-OSEM (Ordered Subset Expectation Maximization) algorithm (24 subsets and 3 iterations), with random, attenuation, and decay correction, and CT was reconstructed with the Modified Feldkamp Algorithm and analyzed using *VivoQuant 4.0* (Invicro Imaging Service and Software, Boston MA) software. Following reconstruction of the images, regions of interest (ROIs) were hand-drawn for select organs using CT images to determine the standard uptake values (SUVs) using VivoQuant imaging software.

### 2.6 Statistical analysis

Data were expressed as mean ± SD. Comparisons were made using GraphPad Prism 9 purchased from GraphPad Software, LLC (Boston, MA, USA) running both ordinary one-way ANOVA and 2-way ANOVA. P values of less than 0.05 were considered significantly different.

## 3.0 Results

### 3.1 Radiolabeling

DOTATATE and DOTA-JR11 were efficiently radiolabeled with > 95% RCY when ~ 5.6 nmol of each ligand was mixed with 3.7 MBq (100 μCi) of [^52^Mn]MnCl_2_ and reaction mixtrure incubated at 90°C for 30 min. The radiolabeling results were confirmed using both the iTLC (free ^52^Mn moves with the solvent front with an R_f_=1 while radiolabeled peptides showed an R_f_=0.36) and HPLC (radiolabeled peptides elute at 10.5 min while free ^52^Mn elute at 2 min) techniques as shown by the chromatograms in Fig. 1.

### 3.2 Serum stability

Serum stability tests of [^52^Mn]Mn-DOTATATE and [^52^Mn]Mn-DOTA-JR11 did not show significant decomplexation 5 days post incubation with mouse serum at 37°C with > 95% intact as assessed by radio-TLC (Fig. 2A). Only less than 10% of activity was associated with the precipitated proteins. HPLC chromatograms of mouse serum stability is provided in Fig. 2B.

### 3.3 Flow cytometry and Cell uptake

Flow cytometry experiments conducted using A549 and MCF-7 cell lines revealed that MCF7 showed roughly 5x lower levels of SSTR2 expression compared to A549 cells (P = 0.008). MCF-7 cells have a lower frequency of SSTR2 + cells, with 0.86% SSTR2 + compared to A549 with a mean of 4.6% (P = 0.0092) (Fig. 3B). Cell uptake studies were conducted to evaluate % cell binding of [^52^Mn]Mn-DOTATATE in AR4J, A549, and MCF-7 cells. The studies showed a significantly higher uptake in AR24J (18.25 ± 1.31% /mg) compared to A549 (4.07 ± 0.88% /mg) and MCF-7 (2.30 ± 0.20% /mg (P < 0.0001) (Fig. 3A) in alignment with the flow cytometry analysis of SSTR2 expression and confirming SSTR2 targeting of [^52^Mn]Mn-DOTATATE.

Comparative cell uptake studies using both [^52^Mn]Mn-DOTATATE and [^52^Mn]Mn-DOTA-JR11 in AR42J and MCF7 revealed different uptake levels (Fig. 4A) with a much higher uptake of [^52^Mn]Mn-DOTATATE in AR24J (11.95 ± 0.71% / mg) compared to the uptake of [^52^Mn]Mn-DOTA-JR11 in AR42J (7.13 ± 0.38%/ mg (P<0.0001)

### 3.4 Cell internalization

Cell internalization assays of both [^52^Mn]Mn-DOTATATE and [^52^Mn]Mn-DOTA-JR11 conducted in AR42J cells revealed that the internalization fraction of [^52^Mn]Mn-DOTATATE was 53.13 ± 1.83% at 2 h while that of [^52^Mn]Mn-DOTA-JR11 was 20.85 ± 0.59%, P < 0.0001(unpaired t test) (Fig. 4B).

### 3.5 PET imaging and biodistribution of [^52^Mn]Mn-DOTATATE and [^52^Mn]Mn-DOTA-JR11 in AR42J tumor bearing mice

Maximum intensity projection (MIP) PET images showed higher tumor uptake of [^52^Mn]Mn-DOTATATE compared to [^52^Mn]Mn-DOTA-JR11 in AR42J tumor-bearing mice, 4 h after injection (Fig. 5). SUV mean analysis of tumor ROIs showed a significant difference in tumor uptake between [^52^Mn]Mn-DOTATATE (1.23 ± 0.22) and [^52^Mn]Mn-DOTA-JR11 (0.09 ± 0.02); P < 0.0001). Other ROIs (Table 1) showed similar results for the two radiotracers.

The systemic distribution of the two radiotracers in AR42J tumor-bearing mice exhibited a comparable biodistribution, with the exception of the uptake in the tumor (Fig. 6). The uptake of [^52^Mn]Mn-DOTATATE in organs such as the stomach, pancreas, small intestines, and liver was: 1.07 ± 0.39, 1.83 ± 0.71, 1.32 ± 0.46, and 0.71 ± 0.16% ID/g, respectively. In comparison, the uptake of [^52^Mn]Mn-DOTA-JR11 in the same organs was found to be 0.90 ± 0.65, 0.39 ± 0.14, 1.23 ± 0.87, and 1.42 ± 0.12% ID/g, respectively, 4 h after injection. The tumor uptake of [^52^Mn]Mn-DOTATATE was significantly higher (11.16 ± 2.97% ID/g (P < 0.0001, 2 way ANOVA)) compared to [^52^Mn]Mn-DOTA-JR11 (2.11 ± 0.30% ID/g) 4 h post injection. The biodistribution profiles correlated well with PET images (Fig. 5), and both radioligands were found to be excreted through the renal pathway. Table 2 provides biodistribution data for the two radiotracers in various organs 4 h after injection. The uptake of [^52^Mn]Mn-DOTATATE in the kidneys was significantly higher (35.96 ± 5.21% ID/g) compared to [^52^Mn]Mn-DOTA-JR11 (19.56 ± 2.09% ID/g).

## 4.0 Discussion

Various somatostatin analogs have been labeled with different PET radionuclides including ^68^Ga, ^18^F, and ^64^Cu to aid in diagnostics, staging, and monitoring treatment outcomes [21]. ^52^Mn has gained significant interest for its positive PET imaging capabilities, including its sufficient positron energy and extended half-life (5.6 d). The extended half-life enables imaging at later time points and facilitates distribution of the radionuclide after production [17]. For this study, we explored the impact of using ^52^Mn as the radionuclide to compare the in *vitro* and in *vivo* effectiveness of the agonist [^52^Mn]Mn-DOTATATE and antagonist [^52^Mn]Mn-DOTA-JR11 in mice bearing AR42J tumors.

The radioligands were successfully prepared with high RCY (> 95%) and specific activities of [^52^Mn]Mn-DOTATATE (0.68 ± 0.05 MBq/nmol) (18.31 ± 1.05 μCi/nmol) and [^52^Mn]Mn-DOTA-JR11 (0.63 ± 0.05 MBq/nmol) (16.89 ± 1.05 μCi/nmol). When incubated with mouse serum at 37°C, the radiotracers showed greater than 95% intact complex over a duration of 5 days similar to stability studies of [^52^Mn]Mn-DOTA reported in previous studies[14, 18]

There is increasing interest in comparing variations of SSTR2 targeting radiopharmaceuticals for molecular imaging and therapy. Reubi et al. showed that the binding affinity, rate of internalization, and tumor uptake of radiolabeled somatostatin receptor 2 agonist analogs can be altered by substitution of the chelator and radiometal. They found that the binding affinity of [^68^Ga]Ga-DOTA-[Tyr^3^]- octreotate in human SSTR2 expressing - CCL39 cells was remarkably higher (IC_50_ 0.2 ± 0.04 nM) compared to [^111^In]In-DTPA-[Tyr^3^]- octreotate (IC_50_ 1.3± 0.2 nM), and [^86^Y]Y-DOTA-[Tyr^3^]- octreotate (IC_50_ 1.6± 0.4 nM) [4, 22]. Recently, Fani et al. also evaluated the effect of radiometal modification on the binding affinity of radiolabled somatostatin antagonists using HEK293-hsst2 cells. In contrast to studies with the agonists, [^68^Ga]Ga-DOTA-JR11(IC_50_, 29 ± 2.7 nM) showed a 60 times lower binding affinity than the respective [^86^Y]Y-DOTA-JR11(IC_50_, 0.47 ± 0.05 nM), and [^111^In]In-DTPA-JR11(IC_50_, 3.8 ± 0.7 nM) [4]. The research groups concluded that the observed differences in binding affinities could be due to the metal coordination geometry differences [4, 22, 23].

In the current work, cell uptake studies demonstrated [^52^Mn]Mn-DOTATATE binding to the SSTR2 in different cell lines with higher uptake in AR42J than MCF7. Initial findings revealed notable variations in cell uptake percentages among AR24J (18.25 ± 1.31% /mg), A549 (4.07 ± 0.88% /mg), and MCF7 (2.30 ± 0.20% /mg) (Fig. 3A). Similar uptake values have been reported by Liu et al. using [^64^Cu]Cu-DOTATATE in MCF7 (0.9 ± 0.06% AD/10^6^ cells) and A549 (0.81 ± 0.21% AD/10^6^) [24]. Cell uptake studies comparing [^52^Mn]Mn-DOTATATE and [^52^Mn]Mn-DOTA-JR11 in AR42J cell line showed that both radiotracers exhibited affinity for SSTR2. However, a higher percentage of cell uptake was observed with [^52^Mn]Mn-DOTATATE (11.95 ± 0.71% / mg) compared to [^52^Mn]Mn-DOTA-JR11 (7.13 ± 0.38%/ mg) after a 2 h incubation at 37°C. In an internalization assay conducted in AR42J cells, it was observed that 53.13± 1.83% of the total activity of [^52^Mn]Mn-DOTATATE was internalized after 2 h incubation. In comparison, only 20.85 ± 0.59% of the total activity of [^52^Mn]Mn-DOTA-JR11 was internalized. In these cell uptake studies, the observed variations revealed the expected differences between agonists and antagonists and align with the trends reported by Rylova et al. They found that after 4 h incubation, 80% of the total activity of [^64^Cu]Cu-DOTATATE was internalized, compared to 60% of the total activity of [^64^Cu]Cu-NODAGA-JR11 in HEK-293 cell line [25]. In a recent study conducted by Xie et al., [^68^Ga]Ga-DOTATATE showed a significantly higher cellular uptake (8.90 ± 0.50% adsorbed dose) compared to [^18^F]AlF-NOTA-JR11 (4.5 ± 0.03% AD) in HEK293-SSTR2 cell line. Additionally, only 5.4 ± 0.32% of [^18^F]AlF-NOTA-JR11 was internalized while [^68^Ga]Ga-DOTATATE showed a much higher internalization rate of 66.89 ± 1.62% after 1 h of incubation [26].

Ahenkorah et al. have reported diverse observations regarding cellular cell uptake, but have obtained comparable results in terms of internalization with both the agonist and antagonist. During their comparative study on QGP1.SSTR2 cells, they examined the membrane binding and internalization of [^18^F]AlF-NOTA-JR11 and [^18^F]AlF-NOTA-TATE. After 1 h of incubation, they observed that [^18^F]AlF-NOTA-JR11 had a binding rate of 85.2 ± 0.9%, with 5.1 ± 0.6% being internalized. In comparison, [^18^F]AlF-NOTA-TATE had a binding rate of 34.9 ± 5.6%, with 23.5 ± 3.6% being internalized. These results deviate from the values we reported in this study, and the variation can be attributed to various factors such as structural changes, modifications in radioelements, and variances in the incubation period. After 240 min, the group discovered that a significant amount of radioactivity of [^18^F]AlF-NOTA-TATE was internalized (> 70%) compared to 10% of [^18^F]AlF-NOTA-JR11 pointing to the effect of incubation time on % internalization [8].

The *in vivo* targeting and biodistribution of [^52^Mn]Mn-DOTATATE and [^52^Mn]Mn-DOTA-JR11 were investigated in AR42J tumor-bearing mice at 4, 24, and 48 h post injection. The tumor showed a significantly higher uptake and retention of [^52^Mn]Mn-DOTATATE (SUVmean: 1.23 ± 0.22) compared to [^52^Mn]Mn-DOTA-JR11 (SUVmean: 0.09 ± 0.02) 4 h after injection. The *ex-vivo* biodistribution results (Table 2, 4h) indicate that the pharmacokinetics of both radioligands are comparable, except for the tumor and kidneys. The tumor uptake of [^52^Mn]Mn-DOTATATE was much higher (11.16 ± 2.97% ID/g) than that of [^52^Mn]Mn-DOTA-JR11(2.11 ± 0.30% ID/g) 4 h post injection. The tumor uptake of [^52^Mn]Mn-DOTATATE decreased over time, with values of (11.16± 2.97% ID/g (4 h), 1.53 ± 0.46%ID/g (24 h), and 0.33±0.13% ID/g (48 h)). In comparison, [^52^Mn]Mn-DOTA-JR11 showed lower uptake and slow washout with values of (2.11 ± 0.30% ID/g (4 h), 0.68 ± 0.11% ID/g (24 h), and 0.26 ± 0.17% ID/g (48 h). Based on the PET images and biodistribution data, it can be observed that the radiotracers are eliminated through the renal pathway. Wadas et al. found similar patterns in their study comparing the tumor uptake of [^64^Cu]Cu-CB-TE2A-BASS (antagonist) and [^64^Cu]Cu-CB-TE2A-Y3-TATE (agonist) in rat pancreatic AR42J xenografts. [^64^Cu]Cu-CB-TE2A-BASS demonstared a slightly lower tumor uptake (1.81 ± 0.56% ID/g) compared to [^64^Cu]Cu-CB-TE2A-Y3-TATE (2.86 ± 0.52% ID/g) 4 h after injection [12]. In a separate study, Xie et al. found that the tumor uptake of [^18^F]AlF-NOTA-JR11 was significantly lower (9.02 ± 5.9% ID/g) compared to [^68^Ga]Ga-DOTATATE (31.35 ± 5.90% ID/g) in mice with HEK293-SSTR2 tumors [26].

In a clinical study comparing [^68^Ga]Ga-DOTATATE and [^68^Ga]Ga-DOTA-JR11 in patients with NETs, Zhu et al. found that [^68^Ga]Ga-DOTA-JR11 had a lower primary tumor uptake (SUVmax: 18.7±17.4) compared to [^68^Ga]Ga-DOTATATE (SUVmax: 32.1 ± 23.7). Despite this difference, both radiotracers showed similar results in patient-based and lesion-based comparisons [7]. In contrast, preclinical studies conducted by Ahenkorah et al. comparaing the PET imaging characteristics between the antagonist [^18^F]AlF-NOTA-JR11 and the agonist [^18^F]AlF-NOTA-TATE in BONI.SSTR2 tumor bearing mice revealed comparable pharmacokinetics and similar tumor uptake of [^18^F]AlF-NOTA-JR11 (SUVmax: 3.7 ± 0.8) and [^18^F]AlF-NOTA-TATE (SUVmax: 3.6 ± 0.4) [8].

In a separate study, the tumor uptake of [^64^Cu]Cu-DOTATATE (20.3 ± 2.5% ID/g) was found to be similar to that of [^64^Cu]Cu-NODAGA-JR11 (20.6 ± 3 .7% ID/g) when the two radiotracers were evaluated in HEK.hsst2 tumor xenografts [25]. It has been observed that in addition to agonistic and antagonistic features, other factors, including the radioelement, tumor type, and chelator, can influence the optimal tumor accumulation [8].

## 5.0 Conclusions

[^52^Mn]Mn-DOTA-TATE and [^52^Mn]Mn-DOTA-JR11 were successfully synthesized with high RCY and purity. Athough both radiotracers demonstrated affinity for SSTR2 receptors, *in vitro* assays showed higher cell uptake and internalization of [^52^Mn]Mn-DOTA-TATE. Through PET imaging, the targeting potential of ^52^Mn labeled probes was revealed, with [^52^Mn]Mn-DOTA-TATE showing higher tumor uptake than [^52^Mn]Mn-DOTA-JR11. The study also confirms that modifying the radiometal can alter the biodistribution and tumor uptake of the radiopetides towards SSTR2. More studies evaluating radiometal- chelator pairs are warranted.

## Figures and Tables

**Figure 1:** Radio-TLC chromatograms for A) free ^52^Mn, B) labeled ligands and C) radio-HPLC chromatograms for blue (free ^52^Mn) and red (labeled ligands)

**Figure 2:** **A)** Stability of [^52^Mn]Mn-DOTATATE and [^52^Mn]Mn-DOTA-JR11 in mouse serum, B) Radio-HPLC chromatograms.

**Figure 3:** A) Comparison of cell uptake of [^52^Mn]Mn-DOTATATE in AR42J, A549, and MCF7. B) Flow cytometry studies showing different levels of SSTR2 expression between MCF7 and A549

**Figure 4:** A) Cell uptake of [^52^Mn]Mn-DOTATATE and [^52^Mn]Mn-DOTA-JR11 in AR42J and MCF7. B) Cell internalization assay of [^52^Mn]Mn-DOTATATE and [^52^Mn]Mn-DOTA-JR11 in AR42J cells

**Figure 5:** PET images of AR42J tumor bearing mice 4 h post injection of A) [^52^Mn]Mn-DOTATATE and B) [^52^Mn]Mn-DOTA-JR11(*n=4*)

**Figure 6:** Biodistribution of A) [^52^Mn]Mn-DOTATATE and B) [^52^Mn]Mn-DOTA-JR11 in AR42J tumor-bearing mice 4, 24 and 48 h after injection.

**Table 1:** SUV mean data of selected organs/tissues extracted form PET/CT images of [^52^Mn]Mn-DOTATATE and [^52^Mn]Mn-DOTA-JR11 in AR42J xenograft models *(n = 4)*

	SUV mean	
Organ/Tissue	[^52^Mn]Mn-DOTATATE	[^52^Mn]Mn-DOTA-JR11
Tumor	1.23 ± 0.22	0.09 ± 0.02
Heart	0.03 ± 0.01	0.03 ± 0.01
Liver	0.11 ± 0.03	0.02 ± 0.01
Kidney	1.55 ± 0.23	0.95 ± 0.01
Muscle	0.02 ± 0.01	0.03 ± 0.01
Bone	0.02 ± 0.01	0.02 ± 0.01

**Table 2:** Biodistribution data (%ID/g) of [^52^Mn]Mn-DOTATATE and [^52^Mn]Mn-DOTA-JR11 in AR42J tumor-bearing mice 4 h after injection (mean ± SD) *n=4*.

Organ/Tissue	[^52^Mn]Mn-DOTATATE	[^52^Mn]Mn-DOTA-JR11
Blood (μL)	0.91 ± 0.05	0.20 ± 0.02
Heart	0.69 ± 0.16	0.92 ± 0.14
Lungs	0.95 ± 0.25	0.49 ± 0.22
Pancreas	1.80 ± 0.71	0.39 ± 0.14
Spleen	0.54 ± 0.14	0.47 ± 0.16
Stomach	1.07 ± 0.39	0.90 ± 0.65
Liver	0.71 ± 0.16	1.42 ± 0.12
Kidney	35.96 ± 5.21	19.56 ± 2.09
Small Intestines	1.32 ± 0.46	1.23 ± 0.87
Large Intestines	5.17 ± 1.39	3.11 ± 1.32
Fat	0.52 ± 0.19	0.18 ± 0.04
Skin	0.83 ± 0.14	0.51 ± 0.20
Muscle	0.19 ± 0.08	0.12 ± 0.02
Femur	0.65 ± 0.27	0.18 ± 0.04
Brain	0.22 ± 0.12	0.10 ± 0.01
Tumor	11.16 ± 2.97	2.11 ± 0.30

## Data Availability

The authors declare that the data supporting the findings of this study are available within the paper. Should any raw data files be needed in another format they are available from the corresponding author upon reasonable request
